# Impacts of Urban Blue-Green Space on Residents’ Health: A Bibliometric Review

**DOI:** 10.3390/ijerph192316192

**Published:** 2022-12-03

**Authors:** Kun Wang, Zhihao Sun, Meng Cai, Lingbo Liu, Hao Wu, Zhenghong Peng

**Affiliations:** 1 School of Urban Design, Wuhan University, Wuhan 430072, China; 2 Wuhan Natural Resources Conservation and Utilization Center, Wuhan 430014, China; 3 Center for Digital City Research, Wuhan University, Wuhan 430072, China; 4 Center for Geographic Analysis, Harvard University, Cambridge, MA 02138, USA

**Keywords:** urban blue-green space, public health, bibliometric analysis, research theme

## Abstract

Urban blue-green space (UBGS), as an important component of the urban environment, is found to closely relate to human health. An extensive understanding of the effects of UBGS on human health is necessary for urban planning and intervention schemes towards healthy city development. However, a comprehensive review and discussion of relevant studies using bibliometric methods is still lacking. This paper adopted the bibliometric method and knowledge graph visualization technology to analyze the research on the impact of UBGS on residents’ health, including the number of published papers, international influence, and network characteristics of keyword hotspots. The key findings include: (1) The number of articles published between 2001 and 2021 shows an increasing trend. Among the articles collected from WoS and CNKI, 38.74% and 32.65% of the articles focus on physical health, 38.32% and 30.61% on mental health, and 17.06% and 30.61% on public health, respectively. (2) From the analysis of international partnerships, countries with high levels of economic development and urbanization have closer cooperation than other countries. (3) UBGS has proven positive effects on residents’ physical, mental, and public health. However, the mediating effects of UBGS on health and the differences in the health effects of UBGS on different ages and social classes are less studied. Therefore, this study proposes several future research directions. First, the mediating effect of UBGS on health impacts should be further examined. Furthermore, the interactive effects of residents’ behaviors and the UBGS environment should be emphasized. Moreover, multidisciplinary integration should be strengthened. The coupling mechanism between human behavior and the environment should also be studied in depth with the help of social perception big data, wearable devices, and human–computer interactive simulation. Finally, this study calls for developing health risk monitoring and early warning systems, and integrating health impact assessment into urban planning, so as to improve residents’ health and urban sustainability.

## 1. Introduction

As the main places of human habitation, cities are the bridge between energy, climate, and mankind, and are one of the most important forms of land use and land cover (LULC) change on the world’s surface [1]. Currently, about 55% of the population lives in cities and towns, and the world’s population is expected to reach 9.7 billion by 2050 [2]. During this period, the population increase will mainly happen in cities, with urbanization expected to reach nearly 70% by 2050 [3]. Climate change and rapid urbanization have led to a series of city-related issues, such as the spread of epidemics, urban flooding, air pollution, urban heat islands, urban noise, and loss of blue and green spaces, which have negatively affected the physical and mental health of urban dwellers [4,5,6,7,8,9]. Over the past few decades, cities have consumed a lot of manpower, material resources, and capital to restore various nature-based forms to reduce the negative impacts of urbanization on human health. 

Urban blue-green space (UBGS) is a strategically planned network of natural and semi-natural areas and other environmental elements, including urban water and vegetation, which can be generalized as urban green infrastructure (UGI) that provides a wide range of ecosystem services within a city [10,11]. UBGS can make great contributions to human health [12]. First, the positive impact of natural landscapes, including blue spaces and green spaces, on the mental health of urban residents has been widely demonstrated [13,14,15,16,17,18,19]. In particular, green space in the streetscape affects mental health more than nearby green space [20]. Second, UBGS motivates residents to increase outdoor physical activity and reduce obesity [21,22,23,24]. At the same time, UBGS is also an important means to prevent the risk of disease infection [25], which can absorb fine particles and other pollutants, improve air quality [26], and reduce noise pollution [27]. The urban heat island effect also brings great stress to the human body, and extreme temperatures can also lead to cardiovascular disease, heat fainting, and heat stroke [28]. UBGS has been widely proven to alleviate the heat island effect [11,28,29,30,31,32,33]. Third, UBGS can increase stormwater infiltration, reduce surface runoff, regulate regional microclimates, and improve water quality [34]. Furthermore, UBGS provides a natural environment for people to interact with each other and prevent loneliness to varying degrees [35]. Finally, the natural landscape of UBGS will improve people’s aesthetic value and comfort [36], and also improve urban biodiversity and promote the harmonious coexistence between humans and nature [37].

UBGS has been recognized by different countries and localities as an important means of promoting residents’ health and well-being. In China, in 2016, the State Council promulgated the “Healthy China 2030 Planning Outline”, which proposed to create a green space environment to promote the health of the people [38]. Moreover, Xie et al. studied the Wuhan East Lake Greenway, the largest urban lake greenway in China, and found that the construction of the East Lake Greenway significantly contributed to the mental health of residents in the surrounding 2 km neighborhood [39]. In the UK, data from nationally representative longitudinal household surveys found that self-reported health and well-being increased with proximity to the coast [40]. Singapore is known as one of the most environmentally friendly cities in the world. According to the park use and satisfaction survey conducted by the Singapore National Park Board in 2009, Zhang et al. concluded that urban green space is positively correlated with mental health [41]. In Plymouth, UK, promoting the central role that green spaces play in contributing to the city’s health, well-being, and biodiversity is among the 2008–2023 green space strategic goals [42]. The stage of UBGS development varies from country to country, as does the degree of health impact. A comprehensive review of the relevant studies in different countries and regions can better promote knowledge in improving human health.

Many studies have concentrated on the impact of UBGS on the health of the residents. [27,43,44,45], but few have been able to draw on a systematic review of this field by using bibliometric and knowledge graph visualization techniques. Quantitative analysis of the literature can provide the latest research progress, the degree of attention and evolution of research hotspots for a certain research field, and can more comprehensively grasp the frontier problems of the research field and judge a country’s scientific status and ability [46]. Therefore, it is necessary to further analyze the research progress and development trend of the effect of UBGS on residents’ health, and to identify research hotspots. To better cope with the challenges of global climate change and urbanization, it is essential to explore the role and mechanism of UBGS in residents’ health and urban resilience.

This study aims to systematically review the literature on the effects of UBGS on residents’ health and provide useful references for research in this field. Bibliometric methods and knowledge graph visualization technology will be adopted to conduct quantitative statistics, data analysis, and knowledge graph visualization of the research literature in the past two decades. The number of publications, international influence, the evolution of keyword hotspots, and the network characteristics of research institutions in this research field will also be explored to reveal the changing patterns of research hotspots during 2001–2021. Based on the identified knowledge gaps, this study will suggest future research directions on the impact of UBGS on residents’ health, urban resilience, and urban planning.

## 2. Data and Methods

### 2.1. Literature Search and Data Processing

The data in this paper were obtained from the Web of Science (WoS) core collection database and China National Knowledge Infrastructure (CNKI), the citation index of WoS was selected from SCIE (Science Citation Index Expanded), and the scope of the study was selected from the literature published in the last two decades (January 2001–December 2021), and the subject terms were limited to “urban green and blue space” OR “urban green space*” OR “urban blue space* “AND” health*” and other titles, abstracts and keywords were searched, WoS and CNKI obtained a total of 5835 English articles and 90 Chinese articles respectively. This extensive search for relevant literature also included studies that were not as relevant to this paper. Therefore, we manually filtered again through the titles and abstracts of the literature, WoS identified 672 English articles and 41 conference articles, and CNKI identified 49 Chinese articles. This information was then used to analyze the evolution of research and current research frontiers on the impact of UBGS on the health of residents.

In this paper, the Bibliometrx R package [47] and CiteSpace [48] were performed for quantitative analysis and knowledge graph visualization. Firstly, Bibliometrx was used to analyze keywords of the literature for co-occurrence, trend analysis of topic words and feature analysis of concept structure, etc., and explore hot and cutting-edge issues. CiteSpace 5.8.R3 was then utilized to measure the burstness detection of keywords for the impact of UBGS on the health of residents during 2001–2021.

### 2.2. Trend and Change-Point Detection

To identify trends and potential points of change in UBGS-related research according to previous research, the Mann–Kendall test and Pettitt’s test were introduced to analyze the publication’s articles from 2001 to 2021 [9,49,50]. The Mann–Kendall test and Pettitt’s test could be performed with the several functions in the R package. The “geo_smooth” function of the “ggplot2” package in the R language program was used to visualize the trend, and the default loess regression method of the “ggplot2” package was used for regression fitting [9,50].

## 3. Results

### 3.1. Changes in the Number of Publications

**Figure 1:**
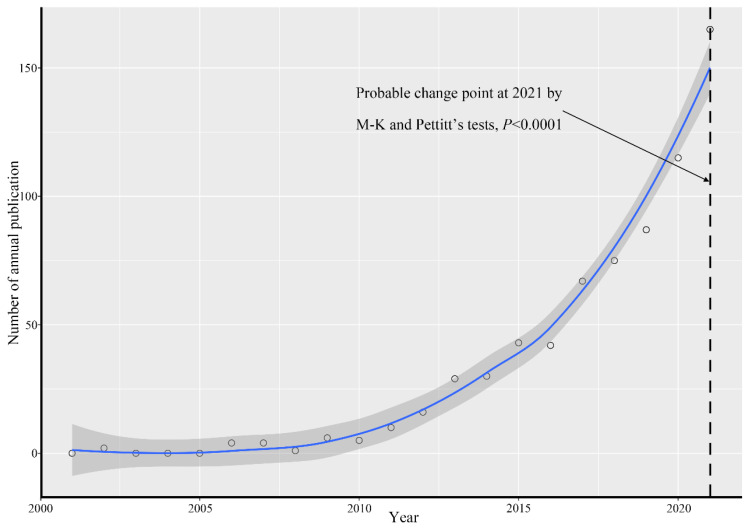
Number of annually published research on the effects of UBGS on residents’ health and change points from Web of Science between 2001 and 2021.

As shown in Table 1 there were 131 sources of literature on the impact of UBGS on residents’ health from 2001 to 2021, mainly including Journals and Books. The average years from publication is 3.97, the average number of citations per paper is 8.429, and the collaboration index is 4.01. Very little material was published during the period from 2001 to 2010, and the number of papers has gradually increased since then. Annual publication trends and change-point detection of articles show a rapid increase in interest in related research, especially since 2021 (change point = 2021, *p* < 0.0001; Figure 1).

**Figure 2:**
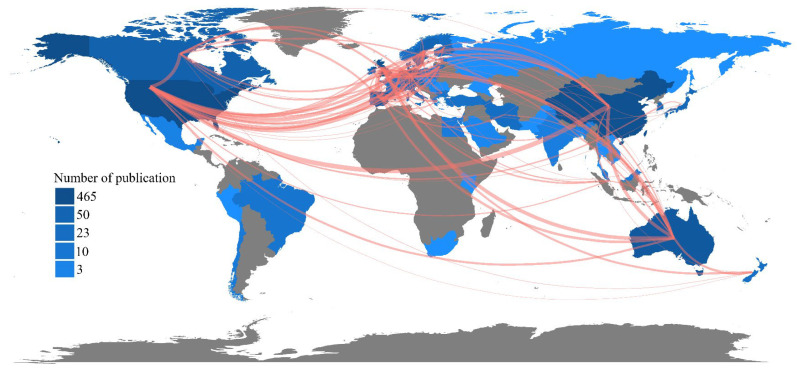
Map of research collaborations in countries around the world from WoS between 2001 and 2021. Thicker lines indicate higher rates of collaboration. Countries that share fewer papers are not shown with connectors.

By the end of 2021, there is a significant imbalance in the amount of literature (Figure 2), with major concentrations in China, the USA, the UK, Australia, and Japan. However, the amount of literature in the field of UBGS’s effect on inhabitants’ health studies in some countries in Africa, South America, and Central Asia is almost zero. Among the country cooperation densities, China and the U.S. have the highest cooperation density, followed by the U.S. and Western European countries, and the U.S. and Australia. It is noteworthy that the density of global research cooperation related to UBGS is mainly concentrated in China, the United States, Australia, and Western Europe, and these three axes of global cooperation density exist.

### 3.2. Research “Hot Topics”

**Figure 3:**
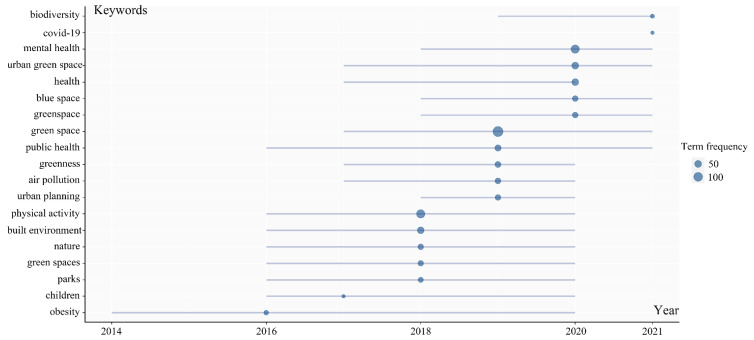
Keywords in UBGS effect on population health from WoS between 2001 and 2021 based on time series clustering distribution. The size of the circles represents the frequency number of keyword occurrences.

**Figure 4:**
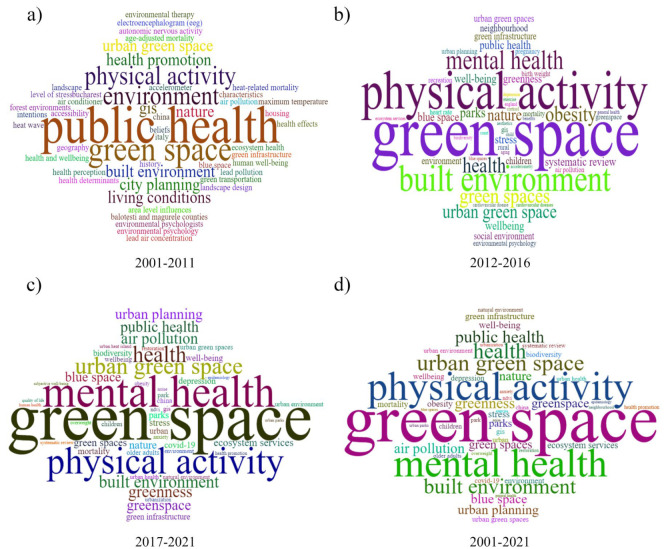
Topic word clouds were detected from keywords in the UBGS-associated publications of Web of Science for different periods between 2001 and 2021. (**a**–**d**) represent the frequency of occurrence in the keywords of the literature on the effects of UBGS on the health of the population for different year ranges, respectively.

**Figure 5:**
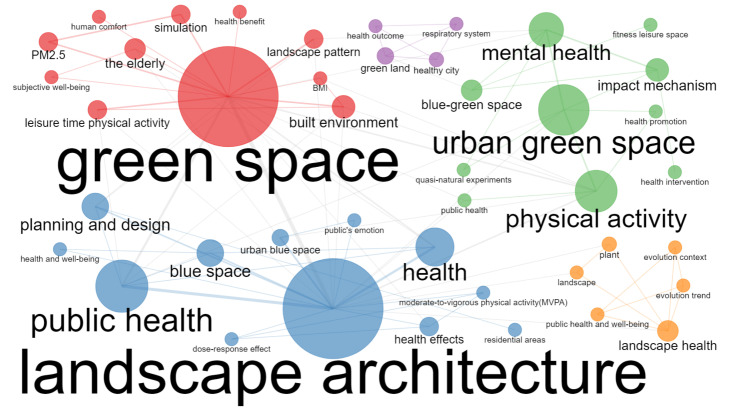
Results of effects of UBGS on residents’ health-related keywords co-occurrence network map from CNKI using Bibliometrix R Package.

A clustering of the 10 most frequently occurring keywords in the literature was performed for further exploration of the “hot spots” in this research field (Figure 3). Most of the research focuses on physical activity, public health, mental health, the built environment, and urban planning. According to the number of articles and keywords in WoS each year, the development process of UBGS’ impact on health can be divided into three stages. From 2001 to 2011, the number of articles published on the impact of UBGS on health was relatively low; from 2012 to 2016, the number of papers published on the impact of UBGS on health increased slowly; from 2017 to 2021, the number of papers published on the impact of UBGS on health increased rapidly (Figure 1 and Figure 4). The concern of UBGS on health is firstly in public health, secondly in physical health, and finally in mental health. The earliest study retrieved from the Web of Science was published in 2002 [51,52]. From 2001 to 2011, the research hotspots were less concerned, and the number of literature was small, mainly focusing on the effect of urban green space on public health, mainly heat waves, air pollution, heat-related mortality, and health perception. Since then, there has been an increasing interest in hot spots of research on the health impacts of UBGS, and various aspects have been analyzed and applied in planning. In China, according to the CNKI database, CNKI mainly focuses on the impact of UBGS on residents’ health with landscape architecture and urban-rural planning disciplines, such as physical activity, mental health, landscape architecture, planning and design, public health, and PM2.5 (Figure 5). Overall, WoS and CNKI databases on the health effects of UBGS are mainly focused on green space, physical activity, mental health, built environment, public health, and urban planning.

### 3.3. Evolution of Multidimensional Research Topics

**Figure 6:**
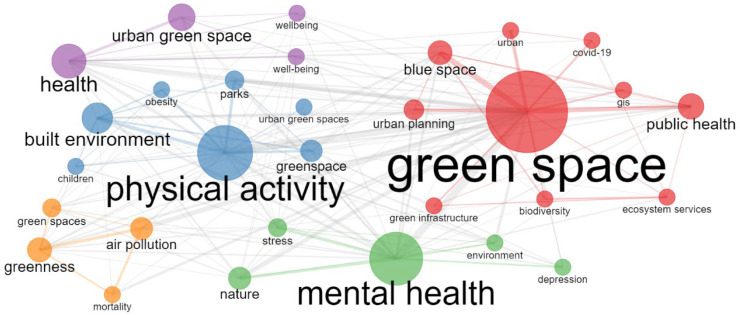
Results of effects of UBGS on residents’ health-related keywords co-occurrence network map from WoS using Bibliometrix R Package.

UBGS directly or indirectly affects the health of residents. Through the co-occurrence network analysis and visualization of keywords in related research, the research progress of the impact of UBGS on residents’ health was explored. After analyzing the keyword co-occurrence network and examining related studies, a meaningful classification was performed. Overall, most of the co-occurrence network of WoS- and CNKI-related research hotspots focused on physical health, mental health, public health, and others (Figure 5 and Figure 6). Among them, based on WoS and CNKI sources related to physical health (*n* = 277, 38.74%; *n* = 16,32.65%), mental health (*n* = 274, 38.32%; *n* = 15, 30.61%), public health (*n* = 122, 17.06%; *n* = 15, 30.61%), and other (*n* = 113, 15.8%; *n* = 9,18.36%) were checked for more extended evaluation. We found that the topic articles on the impact of UBGS on health were counted many times, and the total number of possible classified articles exceeded the number of literature.

#### 3.3.1. Impact of Urban Blue-Green Space on Physical Health

##### Physical Activity

Documenting the many physical activities that UBGS applies to physical health is also a primary topic that we identified. Researchers in this field recognized that different age groups, accessibility of UBGS, and the built environment characteristics of residents all influence physical activity. These studies made it possible to assess their effect on health and happiness and provide public health officials and policymakers to address issues and make decisions.

Much of the effect of UBGS on physical activity health studies relies on green space. For instance, many research literature has focused on the relationship between physical activity and community green space [53,54,55]. Nevertheless, in Scotland, community green space is not related to physical activity, and the influencing mechanism of the relationship between community green space and health may not be explained by physical activity [56]. The impact of urban blue space on physical activity is relatively sparse. The distances and amounts of blue space are significant factors affecting physical activity. Proximity to blue space is related to higher physical activity [57,58,59]. The more blue space there is, the higher the level of physical activity [60,61]. In China, Xie et al.’s research found that the impact of urban greenways on residents’ physical activity has a significant spatial distance attenuation, and the level of moderate to high-intensity physical activity among residents is significantly increased within 1 km [62].

##### Obesity

Thirty-eight papers were concerned with the impact of UBGS on obesity, which is calculated using body mass index (BMI) as measured by an individual’s height and weight, as well as the self-reported height and weight assessments [63]. Studies in Canada [64], Portugal [65], Egypt [66], and New England [67] showed an insignificant relationship between urban green space and obesity. At the community level, street greenery was extracted using street view maps, and studies have shown that street green could have a significant impact on obesity [68,69,70]. Furthermore, some studies have found lower levels of obesity in adults and children living close to blue spaces [71,72]. In China, Sun et al. found that urban green space was not associated with overweight, but slope at the 400 m scale was negatively correlated with BMI [73].

##### Birth Outcomes

Thirty-seven articles studied the association between UBGS and birth outcomes, focusing on the association between UBGS and birth weight (BW), preterm birth (PB), small for gestational age (SGA), and low birth weight (LBW) [74,75]. The normalized difference vegetation index (NDVI), street tree census, and access to main green spaces were used as measures of greenness [76,77], and proximity to the shoreline and freshwater was used as measures of blue space. There is a positive correlation between UBGS and birth outcomes. Green space is protective of birth outcomes, and increasing green space could decrease the risk of negative birth outcomes (e.g., LBW and SGA) [78,79]. Moreover, some studies have shown that residential proximity to freshwater bodies is correlated with higher birth weight [80].

##### Cardiovascular

All of these studies were based on exposure in different residential areas, different age stages (e.g., older and adults), and different genders (e.g., women) of study subjects. Leng et al. [81] found in the Winter City of China that residential green space features were correlated with cardiovascular disease, with residents in neighborhoods with less than 28% green space having a higher risk of hypertension and stroke. A study by Plans et al. [82] in the city of Madrid found an association between green space density within a 1500 m buffer zone and hypertension, particularly significant in women. In addition, among park users, the rates of cardiovascular disease and diabetes were substantially less than among non-users. [83]. Another study reported an increased risk of hypertension in people living in coastal areas [84]. However, between regions, studies have found higher cardiovascular prevalence in those living in continental areas than in those living in coastal areas, especially in women [85].

#### 3.3.2. Impact of Urban Blue-Green Space on Mental Health

##### Self-Reported Mental Health

Twenty-six articles studied the impact of UBGS on self-reported mental health and assessed it using a more validated instrument, including SF-12v2, Kessler-6 Psychological Distress Scale, GHQ12, World Health Organization Well-Being Index (WHO-5), health survey, and census statistics. Urban blue-green spatial data were obtained from remote sensing (e.g., NDVI) and street views. A statistical relationship was established between UBGS and self-reported mental health. Communities in Dutch cities found (N = 223) that residents near green spaces with higher accessibility and availability had higher levels of attachment to local green spaces with higher self-reported mental health [86]. However, studies in Canada found that access to both public green space and overall green space exposure was not significantly correlated with self-reported health status (N = 2,173,170) [87]. For urban blue spaces, living closer to the coast was associated with better self-reported overall health and mental health, but there was no mediating relationship between distance to the coast and health [57]. Consequently, the effect of UBGS on self-reported psychological health was more significantly influenced by subjectivity, regional variability, and gender.

##### Depressive and Anxiety

The impact of UBGS on depressive symptoms and anxiety focuses on older residents and residential surroundings on accessibility to blue-green space. The study in which increased green space around the family was correlated with a decrease in depressive symptoms, but not anxiety. (N = 142) [88]. Gascon et al. found a potentially protective impact of green space on psychological health (depression and anxiety) in adults by generating indicators of blue-green exposure in residential buffers but did not find a significant relationship with blue space. (N = 958) [89]. Research on the Depression Scale for Older Adults in Beijing, China, found that older adults who were exposed to more blue spaces in the streetscape had significantly fewer depressive symptoms (N = 1190) [90]. Nevertheless, research in Barcelona, Spain, showed no association between blue space and depression and anxiety [89].

##### Stress

Stress response to UBGS exposure has been assessed using the Short Form Health Survey (SF-36), General Health SF-12, Perceived Stress Scale, self-reported stress scale, and questionnaire. These studies revealed a positive correlation between UBGS on stress. Stigsdotter et al. revealed that interviewees who were more than 1 km from a green space were 1.42 times more probably to be stressed than those who were less than 300 m from a green space, while those who were not stressed were more often to go to green space than those who were stressed. (N = 21,832) [91]. Roe et al., demonstrated that housing in locations with a large proportion of green space was correlated with lower stress, while there was a substantial adverse association between higher green space and stress. (N = 106) [92]. In Hong Kong, Yang et al. revealed that residential green space cover mitigated the adverse effects of perceived stress on sleep quality (N = 11,954) [93]. In Chicago, Fan et al. found that park green space relieved stress indirectly by promoting social support, while greening of the community had a direct effect on stress relief [94]. At the microscopic scale, Jiang et al., show that higher canopy density in communities increases the control of stress, and in most cases, understory vegetation was negatively correlated with stress [95]. Nevertheless, studies on the effect of urban blue space on stress are relatively sparse. In China, Chen et al., found that urban blue space exposure was significantly correlated and stress relief served as a mediator effect to older adults’ psychological health in the community [96].

#### 3.3.3. Impact of Urban Blue-Green Space on Public Health

##### Air Pollution

All studies show that UBGS has a positive impact on air pollution and that any reduction or increase in the amount of large green space in a city affects the air pollution levels in the cities. However, there is a significant association between air pollution and mortality from different diseases (e.g., respiratory diseases). Jaafari et al., demonstrated that green spaces have a considerable alleviating effect on air pollution and death rates from respiratory diseases in Tehran [97]. However, in adolescents, one study did not observe an association between green space, air pollution, and cardiovascular disease [98]. Li et al., revealed that the adverse association between green space and hypertension fell as air pollution concentrations increased [99]. Urban blue spaces have less impact on air pollution. Hooyberg et al., found lower air pollution concentrations at 0–5 km from the coast, but no statistical association with better health (N = 60,939) [58]. In China, Ding et al., found that the impact of green space landscape on air pollution reduction is more prominent [100].

##### Heat Wave

Twelve articles focus on the impact of UBGS on heat waves. Global climate change and urbanization pose challenges to human health, and more areas of the globe are affected by extreme weather, especially heat waves. Studies have found that heat waves not only cause heat-related diseases such as heat stroke and pyrexia in humans but also increase the risk of death from a variety of cardiovascular and respiratory diseases. Ward et al., studied 70 cities in Europe and found that the size of heat islands in cities and the proportion of green space in urban centers were important influences on heat waves. During heat waves, cities with cooler climates and a higher proportion of urban green space are more vulnerable to the extra heat, northern European countries with cooler temperatures appear to be more vulnerable to heat waves, and southern European countries appear to be better adapted to heat waves [101]. However, at the microscopic scale, the likelihood of mortality during heat waves is higher in areas where the percentage of green space and green roofs around buildings is low [102]. Burkart et al. showed that UBGS had an alleviating effect on heat-related deaths for the aged [103].

##### Epidemic Disease

Environmental characteristics are inseparable from the occurrence and spread of disease. UBGS is an essential ingredient of the city environment, offering multiple ecosystem services that are more important during and after epidemic disease, especially the COVID-19 pandemic. Zhang et al., found a significant adverse effect of the COVID-19 epidemic on mental health, in which the prevalence of mental distress increased 7.84-fold during the epidemic, and the prevalence of UBGS without mental distress was considerably higher than that of those with mental distress [104]. Huang et al., showed a significant positive association between the park and dengue incidence [105].

#### 3.3.4. Other

Finally, we included some classifications of UBGS for health-related studies, but these studies do not fall into one of the specific health categories listed above. The study of the link between UBGS on humankind welfare is a typical one. Reyes-Riveros et al., from the literature suggest that the volume of green space and the scale of vegetation can improve human well-being [106]. Ma et al., researched the effect of urban green spaces on residents’ well-being which showed that the stronger the residents’ involvement in green spaces, the higher their well-being. Furthermore, a significantly inverted U-shaped effect exists between residents’ well-being and the distance to green space [107]. For urban blue spaces, exposure to blue spaces may improve pro-environmental behavior, which can also have a beneficial impact on human health and welfare [108].

### 3.4. Trend of the International Conference

**Figure 7:**
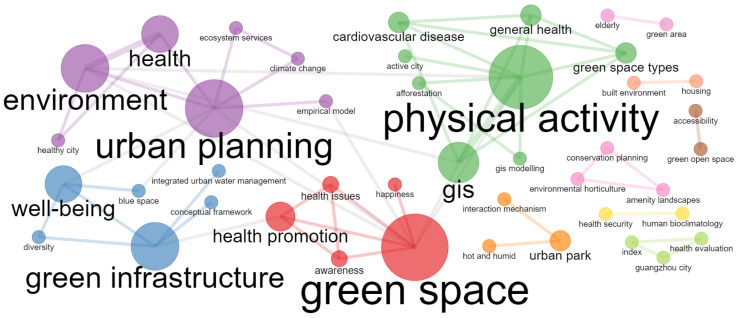
Results of impacts of UBGS on residents’ health-related keywords co-occurrence network map from conference papers using Bibliometrix R Package.

International academic conferences are highly pertinent and cutting-edge for discipline construction and development. It can be seen from the co-occurrence network of keywords related to the impact of UBGS on residents’ health (Figure 7). In the articles of international conferences, the themes of UBGS’s impact on residents’ health present the characteristics of diversity. The co-occurrence network of UBGS on residents’ health-related research hotspots focuses on physical activity, green space, green infrastructure, urban planning, and the environment. In addition, keywords such as climate change, active city, urban park, elderly, health promotion, model, conceptual framework, health evaluation, and interaction mechanism have also deepened the understanding of the impact of UBGS on residents’ health. These keywords involve different environmental behavior theories and methodological research of multidisciplinary integration. Under the interaction of global climate change, urbanization, and artificial intelligence, these research hotspots in the future may make clearer the impact of UBGS on residents’ health.

### 3.5. Conceptual Structure

**Figure 8:**
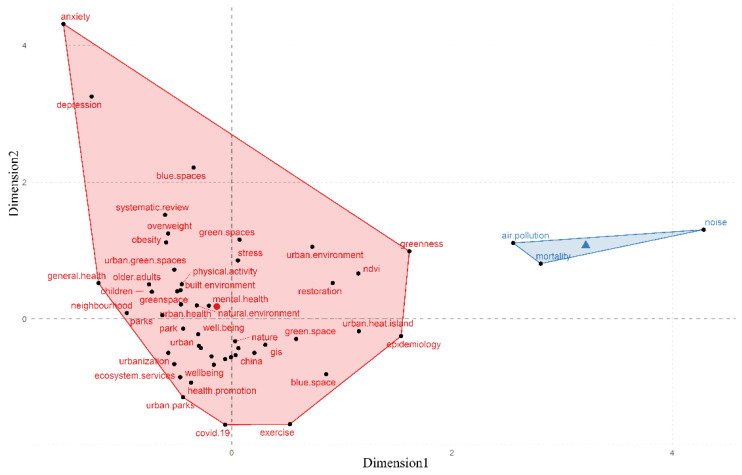
Theme clustering identified from author keywords in UBGS-related publications from Web of Science from 2001 to 2021. The concept structure function of Bibliometrix R software is used to perform multiple correspondence analysis on identified themes and generate graphs and clusters expressing shared topics.

Figure 8 illustrates two clusters of related research keywords on the impact of UBGS on health. One cluster of mostly identified research themes concentrated on the effects of UBGS on physical health (e.g., obesity, overweight, physical activity), mental health (e.g., anxiety, depression, stress), and public health (e.g., COVIDE-19, heat island), and characteristics of the diverse range of ages (e.g., kids, adults, elderly) and regions (e.g., cities, neighborhoods, parks). Another clustering focuses on the areas of association of blue space on health effects, including air pollution, noise, and mortality, and some studies have also shown that UBGS has a positive association with air pollution and noise. In addition, there is a synergistic effect between the impacts of heatwaves and atmospheric pollution on human health, with the two acting together to produce greater health effects and aggravate the risk of death.

Moreover, the vitality of UBGS for health research lies not only in the sustainable development of the natural–economic–social ecological system that has been inherited consistently but also in the constant pursuit of the realistic goal of healthy and livable urban life. The research on UBGS has experienced the transition from the sustainable development of urban natural ecology to the strong sustainable development of urban society. According to the systematic review of articles, urban green space has a greater impact on physical health and mental health, especially mental health, and a large number of literature proves that urban green space has a positive impact on mental health. Urban blue space has a greater impact on public health, especially on heat waves, air pollution, and the urban heat island effect. Furthermore, blue spaces may spend more time outdoors than green spaces, and blue spaces may also improve human well-being more than green spaces. In the context of climate change and urbanization, the impact of UBGS in future cities on public health, mental health, and physical health may be even more profound.

## 4. Discussion

### 4.1. Knowledge Gaps

**Figure 9:**
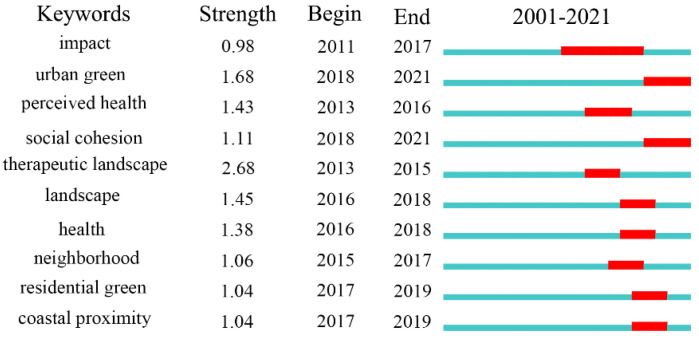
Results of effects of UBGS on residents’ physical health-associated keywords according to the CiteSpace “burstness” test.

UBGS is directly affecting physical health, especially in compact urban areas. Further research is needed to further develop the knowledge of the association between UBGS and health. The effect of UBGS on physical health was considered only for the exposure and accessibility of blue-green space. However, there are no uniform standards for the varying sizes of UBGS buffers, and several studies suggest that the accessibility of UBGS exposure is subject to cultural and climatic differences, which requires further research. Based on Figure 9, the effect of UBGS on physical health in the background of urban green, neighborhood, residential green, and coastal proximity is gaining more and more attention. This indicates that the effect of UBGS on physical health will continue to be carried out to promote sustainable urban development. Since 2008, there has been a significant increase in the study of the ecological service value of UBGS to urban social space, indicating further that research on UBGS on health is transferring from an emphasis on landscape ecosystems to urban social space impacts.

**Figure 10:**
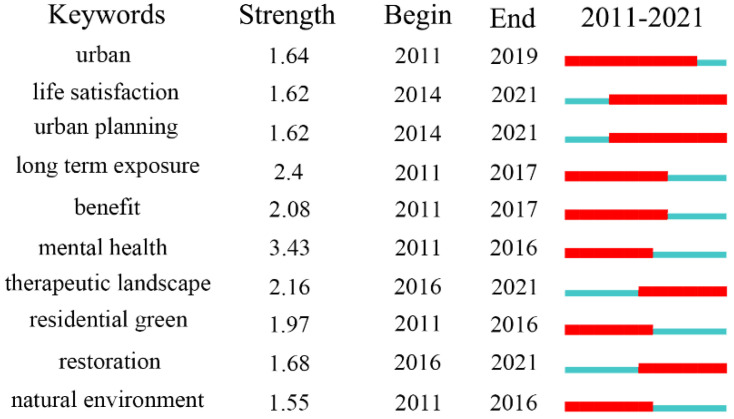
Results of impacts of UBGS on residents’ mental health-associated keywords according to the CiteSpace “burstness” test.

Prior research has indicated that UBGS directly or indirectly affects mental health, and it is an effective hand break to mitigate or alleviate mental illness regulation. However, there are major differences in the moderating effects and mechanisms of action of UBGS on mental health, but there remains a gap in understanding the degree of variation in their moderating effects. By longitudinally studying the impacts of long-term exposure to UBGS on mental health, especially the association of the natural environment, greenery in residential areas, and mental health, it is possible to clarify the difference between the moderating effects of UBGS on mental health between the two and guide the planning response of UBGS on mental health. Figure 10 shows that in the past 10 years, the study of UBGS for mental health has increased, especially in urban planning practices. Blue-green space is a crucial component of nature-based solutions that may promote human health. In street design or limited space, you can improve the green view rate of public space, integrate blue space into urban design, and continuously improve the satisfaction of life.

**Figure 11:**
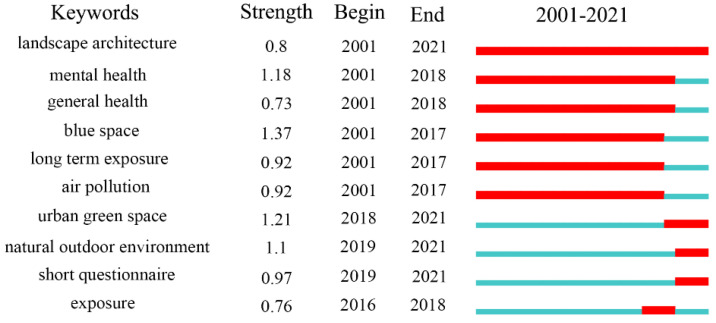
Results of impacts of UBGS on residents’ general health-associated keywords according to the CiteSpace “burstness” test.

In the context of global climate change, extreme weather occurs frequently, and both the high temperature and heat waves generated by climate change, changes in living environments, and epidemic disease transmission vector patterns are due to climate change, all of which pose different degrees of direct or indirect threats to human health. Numerous research has demonstrated that UBGS has a beneficial effect on human health to some extent. However, in the process of urbanization, the availability of UBGS is decreasing and rarely makes up for the lack of UBGS. Although the use of green roofs can increase green space to some extent, the effect of green roofs that can replace UBGS on health is less studied. As can be observed from Figure 11, the effect of urban green space and the natural outdoor environment on general health has received more and more study interest since 2018.

Fewer studies have been conducted on the general health effects of UBGS. A diversity of variables, including air quality, climate, and intensity of human activity can affect general health. Therefore, mediating factors in studies of the impact of UBGS on general health are key to addressing these gaps. The effects of mediating factors such as climate, built environment, airborne particulate matter, vegetation type, and the size range of UBGS on general health in different regions need to be further studied.

### 4.2. Future Prospects

In the context of global climate change and urbanization, further research on the health impacts of UBGS is needed more profoundly. The current long-term survey data have laid the foundation for future longitudinal research on the impact of UBGS on residents’ health. It is helpful to sort out the influence mechanism between UBGS and residents’ health from a microscale. Moreover, future research should pay attention to how different UBGS environmental elements affect residents’ emotions, attitudes, and cognition, and how residents’ psychological factors, cognitive levels, lifestyles, and social characteristics affect the use of UBGS environments. At the same time, the interactive effect of individual behavior and the UBGS environment should also be emphasized.

The impact of UBGS on physical health activities is quite different. Sebastian et al.’s research found that urban green space and public open space have a greater impact on residents’ physical health activities [109]. However, there is a lack of quantitative research on urban blue spaces. Residents spend more time in urban blue spaces than in urban green spaces, and their happiness index is also higher. In addition, future research should also focus on the impact of UBGS exposure ranges in different geographical cultures and climates on the health of different groups of people (such as the elderly and children). Exposure to the imbalance of UBGS exposure in community living affects the health of people in different economic income classes. Wheeler et al. found that urban blue space has better benefits in areas of lower socioeconomic class [110]. Therefore, an interdisciplinary approach should also be undertaken to understand how the use of blue space promotes and limits health.

The influence of UBGS on mental health has been confirmed many times, but there are relatively few studies on the mechanism of the effect of UBGS on mental health. Future research should focus on the mediating effect of UBGS on mental health. In terms of research methods, encouraging long-term longitudinal UBGS exposure investigations on mental health, and horizontal short-term UBGS exposure research on mental health can use wearable devices for physiological, psychological, and behavioral measurements, as well as human–computer interaction scenarios simulation. In terms of research objects, expand the breadth of research objects and evaluation indicators, starting from different age groups and different socioeconomic classes, and continuously improve the evaluation standards and thresholds of UBGS for mental health.

Furthermore, to strengthen multidisciplinary research, the mutual influence and interaction between human behavior perception and environmental elements require the joint promotion of various disciplines and the joint effect of subjective and objective measurement methods. Measure the perception between human behavior and the environment with the help of social sensing big data, wearable devices, and high-performance spatial intelligence human–computer interactive simulation, and use objective tools to evaluate the environmental quality of UBGS. Finally, it is important to pay attention to the negative impact of the UBGS environment on health, especially public health, and UBGS is also a medium for infectious disease attachment.

### 4.3. Integrating Health into the Planning and Application of UBGS

Cities are facing the challenges of urban sprawl and global climate change [11]. Declining urban biodiversity, urban heat islands, urban flooding, and the frequency of climate extremes can all adversely affect residents. However, the quantity and quality of UBGS could provide multifunctional benefits to residents, and their application in urban planning could mitigate these problems in cities, such as alleviating urban heat islands and flooding [111,112,113]. In China, the construction of “sponge cities” has been proposed to address urban flooding and associated urban flood management problems to utilize UBGS for stormwater management and control in the urban environment [113]. Therefore, the planned use of UBGS could enhance the internal permeability and circulation of cities and address local climate and environmental issues [114,115].

Furthermore, UBGS are important components of urban ecosystems, and the application of UBGS in urban planning could best support health and alleviate possible health risks posed by cities [63]. In 2013, the WHO developed the guideline “Adapting to Climate Change to Protect Public Health: Vulnerability and Adaptation Assessment”, which incorporates health risk monitoring and assessment of climate change, in the background of global climate change, into planning and implementation. Under the policy of ecological civilization and implementation of the health China strategy, we should give full play to the resilience of blue-green space to cope with health risks, carry out health risk monitoring and alert and supervision of planning implementation, integrate health impact assessment into urban planning to continuously improve the resilience and adaptability of urban spatial environment.

Finally, to support the application of UBGS in urban planning, several decision systems for supporting planning have been developed [116,117,118]. Nevertheless, UBGS planning is mainly about developing limited spatial networks in compact cities [119], and the potential benefits from UBGS are likely to be only partially utilized, lacking the criteria for planning the multifunctionality of UBGS. Therefore, a systematic spatial assessment of UBGS, socioeconomic, and natural ecological functions should be carried out in the application of urban planning.

### 4.4. Strengths and Limitations of This Review

The current hot spots of research attention are also the focus of continued attention in the future. We also conducted a comprehensive secondary search of the searched relevant literature. Moreover, we only analyzed WoS English articles and CNKI Chinese articles. Consequently, our study could not include the whole document on the effects of UBGS on residents’ health, and some relevant studies may be excluded. However, including Scopus or PubMed scientific database also is not comprehensive, and all have their advantages and disadvantages [120]. Books, reviews, and publications in other languages also offer a wide range of perspectives for studying the effect of UBGS on the residents’ health. In conclusion, this paper compares the general situation and development trend of the publications on the effects of UBGS on residents’ health and provides a theoretical reference for the research related to the impact of UBGS on residents’ health.

## 5. Conclusions

This study uses bibliometric methods and knowledge map visualization technology to systematically review the literature on the impact of UBGS on residents’ health over the past two decades. The number of articles, international influence, global collaboration, and keywords for study hotspots on the health effects of UBGS for the period 2001–2021 have been explored based on 672 English and 49 Chinese articles retrieved from WoS and CNKI. The results show that: (1) The number of articles on the impact of UBGS on the health of residents was on the rise in the last two decades. (2) Countries such as China, the US, and the UK have published the highest number of publications on studies on the effect of UBGS on the health of their residents. The degree of research attention related to UBGS is influenced by different stages of economic development and urbanization levels. (3) Currently, the density of global research collaboration related to UBGS is concentrated in China, the United States, Australia, and Western Europe, and three axes of global collaboration density exist. (4) The co-occurrence network of research hotspots related to UBGS included in WoS and CNKI databases mainly focus on urban green space, green infrastructure, ecosystem services, built environment, urban planning, landscape architecture, physical activity, mental health, and public health. (5) Urban green space has a greater impact on physical and mental health, whereas exposure to urban blue space has a larger impact on human happiness than green space.

While existing research suggests that UBGS is beneficial to some extent, the mediating effects of UBGS on health impacts need to be further explored. Moreover, it is also necessary to explore the differential characteristics of UBGS on the health effects of different age groups and social classes. Moreover, attention should be paid to the interaction between residents’ behavior and the UBGS environment. This study calls for strengthening multidisciplinary integration and adopting social perception big data, wearable devices, and human–computer interactive simulation to deeply study the coupling mechanism between human behavior and the environment.

The findings of this study can also offer several policy implications. In the context of global climate change, urbanization, and the COVID-19 epidemic, it is important to give full play to the resilience of UBGS to cope with health risks, carry out health risk monitoring and alert, and integrating health impact assessment into urban planning, so as to improve residents’ health and urban sustainability. This study therefore suggests the inclusion of UBGS in public health areas and increasing the exposure to UBGS through urban planning policies to improve the health of urban residents.

## Figures and Tables

**Table 1:** Description information for effects of UBGS on residents’ health literature retrieved from WoS. The search for articles originating from WoS in English from 2001 to 2021.

Main Information about Article	2001–2021	Authors	2001–2021	Authors Collaboration	2001–2021
Sources (Journals, Books, etc.)	131	Authors	2626	Articles per author	0.256
Articles	672	Author Appearances	3792	Authors per article	3.91
Average years from publication	3.97	Authors of single-authored articles	16	Co-Authors per articles	5.64
Average citations per article	8.429	Authors of multiauthored articles	2610	Collaboration Index ^1^	4.01

^1^ Note: Collaboration Index was calculated as the total number of authors of multiauthored papers divided by the total number of multiauthored papers.

## Data Availability

Not applicable.
